# The Effects of pH and Excipients on Exenatide Stability in Solution

**DOI:** 10.3390/pharmaceutics13081263

**Published:** 2021-08-16

**Authors:** Alexander Benet, Troy Halseth, Jukyung Kang, April Kim, Rose Ackermann, Santhanakrishnan Srinivasan, Steven Schwendeman, Anna Schwendeman

**Affiliations:** 1 Department of Pharmaceutical Sciences, College of Pharmacy, University of Michigan, 428 Church St., Ann Arbor, MI 48109, USA; abenet@umich.edu (A.B.); jukyung@umich.edu (J.K.); aprilkim@umich.edu (A.K.); roseack@umich.edu (R.A.); schwende@umich.edu (S.S.); 2 Department of Medicinal Chemistry, College of Pharmacy, University of Michigan, 428 Church St., Ann Arbor, MI 48109, USA; thalseth@umich.edu; 3 Xiromed, 180 Park Ave, Suite 101, Florham Park, NJ 07932, USA; Santhana.srinivasan@xiromed.com; 4 Biointerfaces Institute, National Community Reinvestment Coalition, 2800 Plymouth Rd, Ann Arbor, MI 48109, USA; 5 Department of Biomedical Engineering, University of Michigan, 2200 Bonisteel Blvd, Ann Arbor, MI 48109, USA

**Keywords:** exenatide, generic peptides, GLP1 agonists, diabetes, analytical characterization, physical/chemical degradation

## Abstract

Exenatide, a glucagon-like peptide-1 receptor agonist, is the active pharmaceutical ingredient in Byetta^®^ and Bydureon^®^, two type 2 diabetes drug products that have generics and multiple follow-up formulations currently in development. Even though exenatide is known to be chemically and physically unstable at pH 7.5, there lacks a systematic evaluation of the impact of pH and excipients on the peptide solution stability. In this study, we established analytical methods to measure the chemical and physical degradation of the peptide in solution. Exenatide remained relatively stable at pH 4.5 when incubated at 37 °C. At pH 5.5–6.5, degradation was driven by oxidation, while driven by deamidation at pH 7.5–8.5. Significant aggregation of exenatide at pH 7.5 and 8.5 was detected by size exclusion chromatography and dynamic light scattering. Each pH value greater than 4.5 exhibited unique profiles corresponding to a loss of α-helical content and an increase in unordered structures. The addition of sugars, including mannitol, sorbitol and sucrose, conferred small protective effects against peptide aggregation when incubating at pH 7.5 and 37 °C, as measured by size-exclusion chromatography and dynamic light scattering. The results of this study will be useful for investigators developing generic exenatide products, peptide analogs and novel exenatide drug delivery systems.

## 1. Introduction

Exenatide, also known as exendin-4, is a 39-amino acid glucagon-like peptide-1 (GLP-1) receptor agonist that acts as an incretin mimetic for the treatment of type II diabetes mellitus [1]. Exenatide retains 53% homology with endogenous human GLP-1 while having several amino acid additions and substitutions that give rise to its increased serum half-life and potency [2,3,4,5]. Exenatide has a partially-folded tryptophan (Trp) cage that prevents degradation by NEP 24.11, the main peptidase responsible for GLP-1 breakdown in vivo [2]. Exenatide is the active pharmaceutical ingredient found in AstraZeneca’s Byetta^®^, a solution formulation for twice-daily injection, and the more successful Bydureon^®^, which consists of exenatide encapsulated in poly(lactide-co-glycolic acid) (PLGA) microspheres for weekly injection [6,7]. In 2020, Byetta^®^ and Bydureon^®^ sales reached over $68 and $448 million, respectively, in a highly competitive GLP-1 product field [8]. There are no FDA-approved generics of Byetta^®^ or Bydureon^®^ currently available. Teva Pharmaceuticals reached an agreement with AstraZeneca allowing them to manufacture and commercialize a generic version of Byetta^®^ as of October 2017 but has yet to reach the market due to its pending ANDA FDA approval [9,10,11]. Aside from generics, ongoing research is geared towards the development of novel controlled-release formulations, such as hydrogels and nanospheres, as alternatives to Bydureon^®^ [1,2,12,13,14]. Some studies examining the stability of exenatide released from PLGA microspheres can be found in the literature [12,15,16,17].

A recently published FDA guidance on ANDA submissions for generic synthetic peptides highlights the importance of characterizing product-related impurities that may affect their safety, immunogenicity and effectiveness [18]. Five peptide products are specifically mentioned, including glucagon, liraglutide, nesiritide, teriparatide and teduglutide. Additionally, the guidance specifically states that characterization should include elucidation of primary sequence and identification of chemical impurities, physicochemical properties and oligomer/aggregation states. Recent studies had shown that microclimate pH in PLGA microspheres containing exenatide increases from acidic to neutral value following in vivo administration, highlighting the importance of investigating pH-dependent degradation of exenatide for the development of generic versions of Bydureon^®^ [19].

Thus, understanding the chemical and physical stability of exenatide is critical to the development of potential generic exenatide drug products and novel extended-release formulations. Identifying the peptide’s potential mechanisms of degradation allows for the optimization of formulation and selection of appropriate manufacturing conditions to avoid the formation of product impurities [20,21]. At present, no systematic studies are available in the literature investigating exenatide’s solution stability. Based on exenatide’s sequence, several deamidations and oxidation hot spots are evident, as highlighted by the following underlined peptide residues (HGEGT-FTSDL-SKQME-EEAVR-LFIEW-LKNGG-PSSGA-PPPS). Exenatide contains two likely deamidation sites, N28 and Q12, as well as two likely oxidation sites, M14 and W24. Only some of these chemical impurities are identified and noted in the formulation literature. [14,16] Structurally, exenatide contains three major domains, including an N-terminal unordered, hydrophilic strand (residues 1–10), an α-helical coil (residues 11–28) and a C-terminal hydrophobic, proline-rich, partially-folded Trp-cage (residues 29–39) [22]. It was reported that Trp-cage disruption is likely responsible for the physical degradation of exenatide [22]. While a variety of isolated analytical methods for the characterization of exenatide’s purity have been mentioned in regulatory and peer-reviewed literature, an analysis of its impurities and their underlying degradation mechanisms is missing [22,23]. As such, the goals of this study were to identify and characterize exenatide’s chemical and physical degradation through the use of accelerated stability testing to elucidate exenatide’s underlying degradation mechanisms.

## 2. Materials and Methods

### 2.1. Materials

Exenatide powder was generously provided by Amneal^®^ Pharmaceuticals (Ahmedabad, India). All other materials were purchased from commercial suppliers.

### 2.2. Exenatide Incubation Conditions

Exenatide solutions were prepared at a concentration of 0.5 mg/mL in various 30 mM buffer solutions according to their respective buffer capacities, including sodium acetate (pH 4.5), sodium citrate (pH 5.5), sodium phosphate (pH 6.5), HEPES (pH 7.5) and HEPBS (pH 8.5). To investigate the impact of excipients, salt (154 mM NaCl) or sugars (4.3% *w*/*v* mannitol, sorbitol or sucrose) were dissolved in 30 mM HEPES buffer (pH 7.5). These solutions were then used to reconstitute exenatide powder. Excipient samples were compared against exenatide reconstituted in 30 mM acetate buffer (pH 4.5) containing 4.3% (*w*/*v*) mannitol, the negative control. Samples were incubated at 37 °C for 30 min to induce conformational equilibrium. Reconstituted exenatide samples at various pH and excipient conditions were subjected to 4 weeks of incubation at 37 °C, removed from the incubator at 0, 1, 2 and 4 weeks and immediately analyzed. No significant changes in pH were observed over the incubation.

### 2.3. Reverse Phase Liquid Chromatography

Exenatide and its chemical degradation impurities were separated by hydrophobicity by reverse-phase liquid chromatography on a C4-Pack column (YMC, Devons, MA, USA) with a Waters 2595 Alliance System (Waters, Milford, MA, USA) on interfaced to a 2995 Photodiode Array Detector (Waters, Milford, MA, USA). Samples were filtered and injected at a concentration of 0.125 mg/mL at a volume of 50 µL and delivered using a mobile phase of acetonitrile (ACN) (0.1% TFA)/H_2_O (0.1% TFA) at a flow rate of 1 mL/min, with a gradient ramping from 30 to 55% ACN (0.1% TFA) over the course of 25 min. The column temperature was held at 40 °C. Exenatide samples were detected from UV absorbance chromatograms that were extracted at 280 nm.

### 2.4. Impurity Identification by Liquid Chromatography with Mass Spectrometry

Exenatide chemical degradation impurities were separated by reverse-phase liquid chromatography on the same C4-Pack column and analyzed by a dual electrospray ionization equipped Agilent 6520 Accurate-Mass Q-ToF (Agilent, Santa Clara, CA, USA). Samples were filtered and injected at a concentration of 0.125 mg/mL at an injection volume of 20 µL and delivered using a mobile phase of ACN (0.05% TFA)/H_2_O (0.05% TFA) at a flow rate of 0.3 mL/min with a gradient ramping from 30 to 55% ACN (0.05% TFA) over the course of 25 min. The column temperature was also held at 40 °C. The MS parameters included a capillary voltage between 1.2 and 2.0 kV, a ToF-MS range of 300–3200 *m*/*z*, a drying-gas temperature of 325 °C, a drying-gas flow rate of 12 L/min, a nebulizer pressure of 45 psi, and a fragmentor voltage of 225 V. The total ion chromatogram (TIC) was detected at 280 nm, with impurity peak mass extractions generated by Qualitative Analysis Mass Hunter Software version b0705 (Agilent, Santa Clara, CA, USA).

### 2.5. Size Exclusion Chromatography

Exenatide physical degradation impurities were separated by molecular weight using size exclusion chromatography on a Superdex Increase 75 10/300 GL column (GE Healthcare, Chicago, IL, USA) with a Waters 2707 autosampler (Waters, Milford, MA, USA) connected to a 1525 binary HPLC pump (Waters, Milford, MA, USA), interfaced with a 2489 UV/Vis detector (Waters, Milford, MA, USA). Samples were filtered and injected at a concentration of 0.125 mg/mL at a volume of 50 µL and isocratically delivered with a pH 7.4 PBS mobile phase at a flow rate of 1 mL/min over the course of 25 min. Molecular weights of exenatide peaks were determined from a standard calibration, generated from the injection of Uracil (MW 114 Da), Aprotinin (MW 6.5 kD), Cytochrome C (MW 14.5 kD), Carbonic Anhydrase (MW 19.5 kD) and BSA (MW 65 kD). Maximum peak height retention times for each standard (t_R_s) were correlated with their MWs, imported onto a semi-log plot with the generated equation, that were used to estimate the MW of exenatide monomer and aggregate peaks (not shown). Exenatide samples were detected from UV absorbance chromatograms that were extracted at 280 nm.

### 2.6. Particle Size Distribution by Dynamic Light Scattering

Particle size distributions were determined by dynamic light scattering on a Zetasizer ZSP Nano (Malvern Panalytical, Malvern, UK). A total of 100 µL aliquots of undiluted exenatide (0.5 mg/mL) were placed into low-volume TruView cuvettes (Biorad, Hercules, CA, USA) at an undiluted concentration of 0.5 mg/mL and analyzed. Attenuation and accumulation numbers were automatically optimized for each sample by the instrument prior to analysis. Particle size distributions were reported by volume and combined into 0.3–10, 10–100, 100–500, 500–1000 and 1000+ nm size ranges to match our instrument’s limit of detection specification ranges [24].

### 2.7. Intrinsic Fluorescence

Intrinsic fluorescence was used to determine the exenatide’s tertiary structure. Undiluted exenatide samples were placed into a QS 1.5 mm quartz cuvette (Hellma, Mullheim, Germany) and analyzed at a concentration of 0.5 mg/mL on a SpectraMax M3 (Molecular Devices, San Jose, CA, USA) plate/cuvette reader. The fluorescence was measured using a wavelength emission range of 280–450 nm and an excitation wavelength of 270 nm while obtaining fluorescence at 6 flashes per reading.

### 2.8. Far-UV Circular Dichroism

Circular dichroism was used to determine exenatide’s secondary structure. Exenatide samples were analyzed at a concentration of 0.125 mg/mL on a J-815 (JASCO, Easton, MD, USA). Instrument sample temperature was held at 37 °C using a Peltier attachment. Circular dichroism spectra were averaged from 5 collected scans over a wavelength range of 245 to 195 nm, with an accumulation rate of 20 nm/min and a data integration time (DIT) of 4 s. Percent contributions from α-helix, β-sheet and unordered secondary structures were quantified using a third-party Spectra Manager Suite (JASCO) add-in, CDPro analysis. In this software, the CONTIN method was chosen, using soluble-membrane protein 56 (SMP 56) as the reference protein for secondary structural analysis.

## 3. Results

### 3.1. Identification of Degradation Impurities by Liquid Chromatography-Mass Spectrometry (QToF)

**Figure 1:**
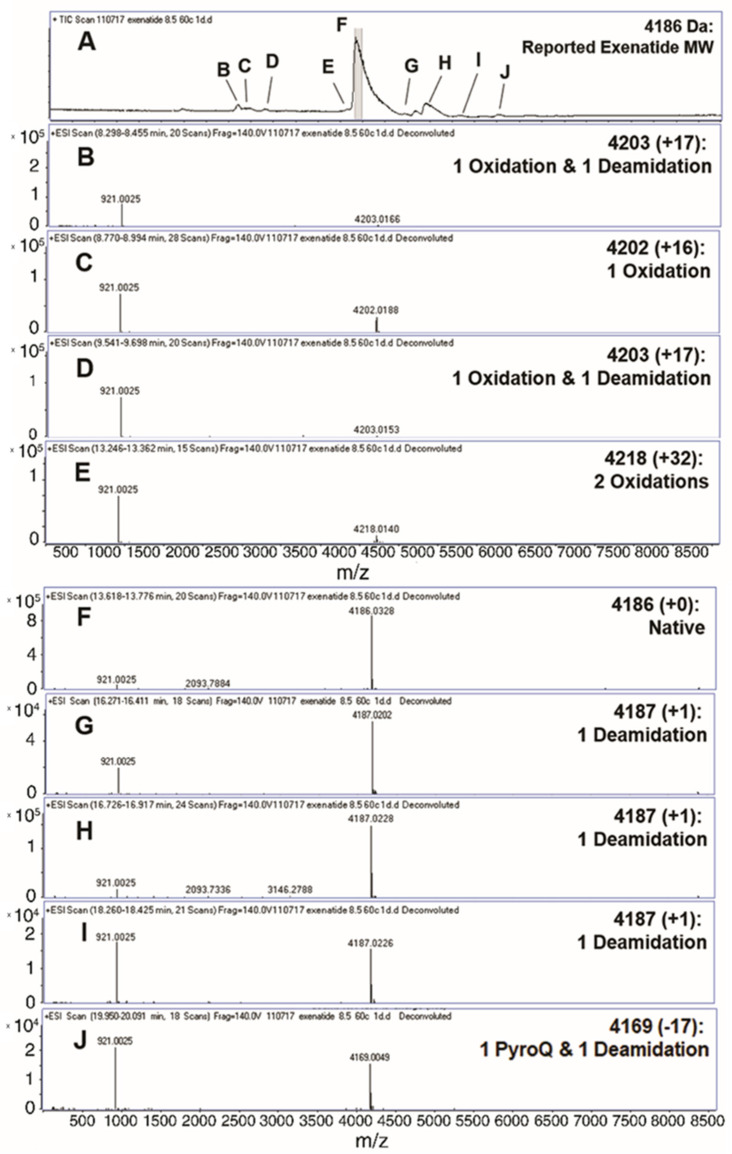
Identification (with deconvoluted masses) of exenatide’s chemical impurities by reverse-phase liquid chromatography-mass spectrometry (QToF) following incubation (pH 8.5) at 60 °C for 24 h. Reverse phase liquid chromatography C4 UV absorbance (**A**) total ion chromatograms and (**B**–**E**,**G**–**J**) exenatide chemical impurities and their reported respective mass shifts, which include a combination of oxidation, deamidation and PyroQ impurities, from (**F**) native exenatide’s MW of 4186 Da.

Liquid chromatography-mass spectrometry (QToF) was implemented to analyze the formation of exenatide chemical impurities. A short-term study was performed by incubating exenatide, reconstituted at pH 8.5, at an elevated temperature of 60 °C for 24 h. Figure 1A depicts the total ion chromatogram with respective peak identities. A total of nine different peaks were identified (Figure 1B–J), including oxidations, deamidations and PyroQ formation. Peak F was identified as exenatide’s native state, parent peak (+0); peaks C and E, single (+16) and double oxidations (+32), respectively; peaks B and D, a combination of oxidation and a deamidation (+17); peaks G, H, and I, a single deamidation (+1); and peak J, PyroQ formation from glutamine (Q12) with a deamidation (−17). A previously published study has confirmed N28 as the primary deamidation site for exenatide when incubated at pH 7.4 for 56 days at 37 °C [25]. In addition, deamidation at the N/G residue combination is prolific in peptides and proteins [21,26,27]. The major deamidation impurity (Peak H) was previously identified as the conversion to L-isoAspartate (L-isoAsp), while the minor impurity (peak G) was identified as the conversion to D-isoAspartate (D-isoAsp) [25]. We would expect that the observed oxidation is occurring at Met14, another primary degradation hotspot implicated to affect peptide and protein stability [21,26,27,28]. Additional tandem mass spectrometry studies are needed to identify specific oxidation impurity locations.

### 3.2. Forced Chemical Degradation at 37 °C

**Figure 2:**
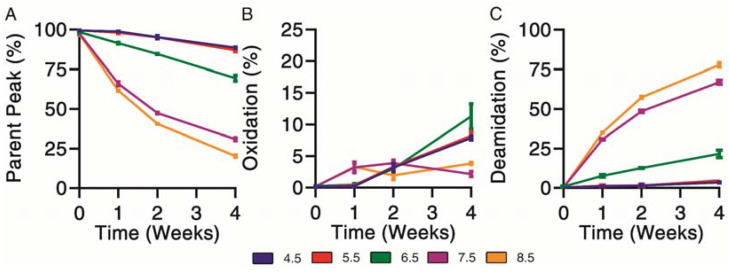
Kinetics of chemical degradation determined by reverse-phase liquid chromatography separating (**A**) parent peak from (**B**) oxidation and (**C**) deamidation chemical impurities during incubation of exenatide solutions pH 4.5–8.5 at 37 °C for 4 weeks. (*n* = 3, mean ± SEM).

Chemical degradation profiles were determined for exenatide solutions reconstituted between pH 4.5–8.5 that were subject to incubation at 37 °C for 4 weeks. Exenatide’s parent peak was separated from its chemical impurities on a reverse-phase liquid chromatography column. Parent peak (Figure 2A), oxidation (Figure 2B) and deamidation (Figure 2C) impurities were quantified over the course of incubation. When incubated at pH 4.5 and 5.5, the parent peak remained relatively stable, decreasing to 88.6 ± 0.7% and 87.0 ± 1.4%, after 28 days of incubation, respectively. The relative stability of exenatide at low pH was expected as Byetta^®^ is commercially formulated in an acetate buffer solution at pH 4.5. On the other hand, rapid, pH-dependent degradation of exenatide was observed at pH 6.5, 7.5 and 8.5. We also observed a loss of total AUCs, indicating some precipitation occurring at pH 7.5 and 8.5 after 2 and 4 weeks, decreasing to 71.9 ± 10.1% and 70.9 ± 9.8% relative to day 0, respectively (Supplementary Material Figure S1B).

Chemical degradation appeared mainly oxidation driven from pH 4.5 to 6.5. As previously mentioned, we would expect this to occur at the methionine residue. Oxidation was identified by the presence of a split peak around a retention time of 6.5 min, where total oxidation was quantified by the sum of peaks eluting prior to the parent peak (Supplementary Material Figure S1A). Over the incubation, the chemical degradation of exenatide at pH 7.5 and 8.5 appeared mainly deamidation driven after 2 and 4 weeks, with the parent peak decreasing to 30.9 ± 1.6% and 20.3 ± 1.1%, respectively, and the deamidation impurities increasing to 67.0 ± 2.2% and 77.8 ± 1.9%, respectively. Again, we expect that the formed deamidation impurity occurs at the N28 residue, as it is followed by a glycine, forming L-isoAsp (at 12.4 min) and D-isoAsp (at 12.4 min) [21,26]. Both isoAsp impurities were previously reported to exhibit weaker GLP1 receptor binding [25].

### 3.3. Forced Physical Degradation at 37 °C

**Figure 3:**
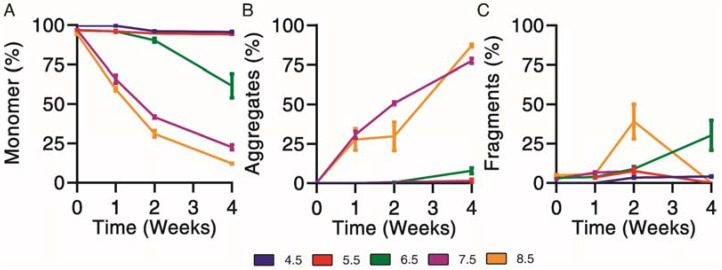
Kinetics of physical degradation determined by size exclusion chromatography, separating (**A**) monomer from (**B**) aggregate and (**C**) fragment physical impurities of exenatide reconstituted at pH 4.5–8.5 after incubation at 37 °C for 4 weeks. (*n* = 3, mean ± SEM).

Physical degradation profiles were determined for exenatide solutions reconstituted between pH 4.5–8.5 that were subject to incubation at 37 °C for 4 weeks. First, size exclusion chromatography was used to separate and quantify monomer (Figure 3A), aggregate (Figure 3B) and fragment (Figure 3C) impurities over the course of incubation.

Physical degradation, indicated by monomer loss, appeared to occur in a pH-dependent manner. Exenatide incubated at pH 4.5 and 5.5 remained relatively stable after 2 and 4 weeks of incubation, with monomers decreasing to 95.7 ± 0.8% and 94.2 ± 0.1%, respectively (Figure 3A). Again, the relative stability of exenatide at low pH was expected as Byetta^®^ is commercially formulated at pH 4.5. Physical degradation tendencies varied significantly at pH 6.5 and above in terms of aggregation and fragmentation profiles (Supplementary Material Figure S1C). Exenatide reconstituted at pH 6.5 tends to form fragments after 2 and 4 weeks of incubation, increasing to 9.0 ± 2.5% and 30.3 ± 16.4%, respectively, rather than aggregates, which increased slightly to 0.6 ± 0.2% and 8.0 ± 1.9%, respectively. On the other hand, exenatide reconstituted at pH 7.5 formed mainly aggregates after 2 and 4 weeks of incubation, increasing to 50.6 ± 1.8% and 77.4 ± 2.9%, respectively. After 4 weeks of incubation, the total AUCs at pH 7.5 and 8.5 decreased, reaching 61.6 ± 0.3% and 61.1 ± 12.1% of the initial values, respectively, indicating some precipitation occurring (Supplementary Material Figure S1D).

### 3.4. Characterizing Particle Size Distribution by Dynamic Light Scattering

Particle size distributions were determined by dynamic light scattering to further confirm the pH dependence of physical degradation and oligomer formation. Particles sizes were measured by volume due to the non-spherical nature of exenatide. Particles were combined into 0.3–10, 10–100, 100–500, 500–1000 and 1000+ nm size ranges. Dynamic light scattering has high run-to-run variability and disproportionally high contribution of larger particle sizes to the overall light scattering signal. Thus, the data do not represent the actual percentages of formed oligomers and are only considered for qualitative purposes. Particle size distributions over the course of 4 weeks at 37 °C are shown in Table 1. At pH 4.5 and 5.5, particles were observed primarily in the 0.4–10 nm size range over the incubation. At pH 6.5 and above, there was a noticeable reduction of particles in the 0.3–10 nm size range that was matched by an increase in particles in the 10-100 and 100+ nm size ranges, markedly shifting at pH 7.5 and completely shifting at pH 8.5, which seem to corroborate the trends of oligomer formation indicated by decreases of total AUC observed by size exclusion chromatography and reverse-phase liquid chromatography.

### 3.5. Structural Analysis by Intrinsic Fluorescence and Circular Dichroism

**Figure 4:**
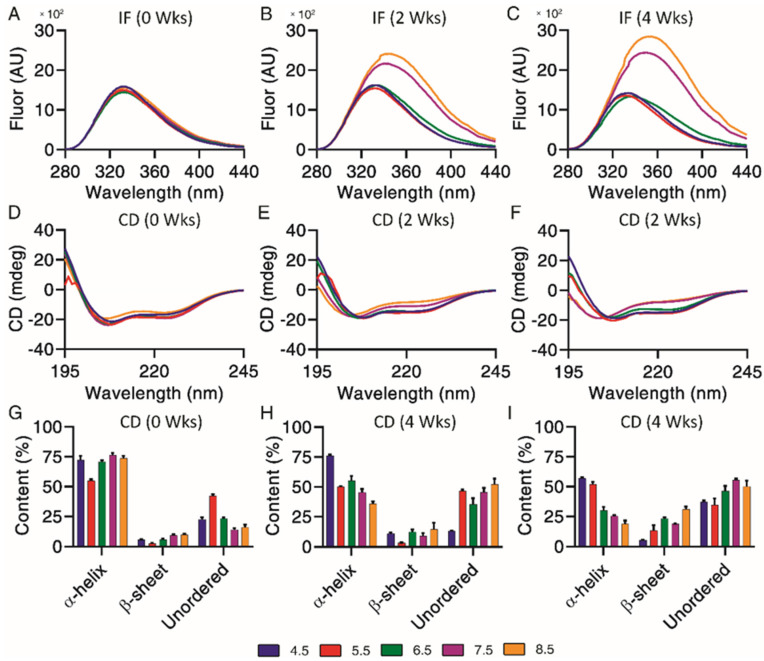
Structural and conformational changes characterized and measured by (**A**–**C**) intrinsic fluorescence and (**D**–**F**) circular dichroism, with secondary structures (**G**–**I**) quantified by CDPro analysis (*n* = 3, mean ± SEM) for exenatide that was reconstituted at pH 4.5–8.5 after incubation at 37 °C for 4 weeks.

Underlying structural changes are important when investigating degradation profiles, as they provide more insight into the impact of chemical and physical impurities. Intrinsic fluorescence was used to qualitatively investigate changes of exenatide’s tertiary structure over the incubation, where both increases of fluorescence and a right shift of maximum emission wavelengths (red shift) were indicators of unfolding through increased solvent exposure of Trp (Figure 4A–C). Exenatide’s single Trp is buried in a proline-rich C-terminal cage that stabilizes exenatide.22 Initially, intrinsic fluorescence profiles were very similar for all pH conditions in terms of intensities (~1500 units) and maximum emission wavelengths (333 nm) (Figure 4A). The intrinsic fluorescence profiles for exenatide at pH 4.5 and 5.5 remained similar over the incubation, retaining maximum emission wavelengths of 333 nm (Figure 4B,C, respectively). For exenatide at pH 6.5, there was an observable right shift of maximum emission wavelength to 341 nm, though with a decrease in fluorescence intensity (Figure 4C). After 2 and 4 weeks of incubation at pH 7.5, exenatide exhibited a large fluorescence intensity increase to 2150 and 2425 units, respectively, while exhibiting a right shift of maximum emission wavelength to 342 and 348 nm, respectively (Figure 4B,C, respectively). An even more marked change was observed for exenatide reconstituted at pH 8.5 after 2 and 4 weeks of incubation, where fluorescence intensity increased to 2390 and 2835 units, respectively, while maximum emission wavelengths shifted to 344 and 354 nm, respectively (Figure 4B,C). Both an increase in intrinsic fluorescence and shifting of max emission wavelength indicated Trp cage unfolding.

Circular dichroism is useful in combination with intrinsic fluorescence, providing further information on changes of underlying secondary structural content, including α-helical, β-sheet and unordered content. Exenatide samples were qualitatively measured by circular dichroism spectra (Figure 4D–F) and then quantified by CDPro analysis (Figure 4G–I). Initially, base emission wavelengths were similar for all pH conditions, at 208 nm (Figure 4D). Quantified secondary structures were also initially similar for all pH conditions, apart from pH 5.5 (Figure 4G). The base emission wavelength for exenatide reconstituted at pH 4.5 remained unchanged, at 208 nm, over the course of incubation, though exhibiting a loss of α-helical content (72.3 ± 6.1% at 0 weeks vs. 57.1 ± 1.6% after 4 weeks) and increase in unordered content (22.7 ± 2.8% at 0 weeks vs. 37.1 ± 2.1% at 4 weeks). Exenatide reconstituted at pH 5.5 also retained a base emission wavelength of 208 nm. While having a lower initial α-helical content, the secondary structure remained unchanged over the course of incubation. The α-helical content for exenatide reconstituted at pH 6.5, 7.5 and 8.5 decreased in a pH-dependent manner from 70.9 ± 2.2%, 76.6 ± 2.6% and 73.7 ± 3.4% at week 0, respectively, to 30.1 ± 5.1%, 25.4 ± 1.3%, and 18.8 ± 4.0% after 4 weeks, respectively (Figure 4G–I). At pH 6.5 and above, the loss of α-helical content was generally matched by an increase in unordered and some β-sheet content.

### 3.6. The Impact of Excipients on Degradation at pH 7.5

Sugar excipients are generally known to offer protection against aggregation during freeze/thaw cycles and long-term storage, while other excipients, such as salt, may induce aggregation [21,26]. The impact of these excipients on chemical and physical degradation profiles was investigated. Exenatide at pH 7.5 in the presence of various excipients was compared against exenatide reconstituted at pH 4.5 with 4.3% mannitol, the negative control (mimicking Byetta^®^ formulation).

**Figure 5:**
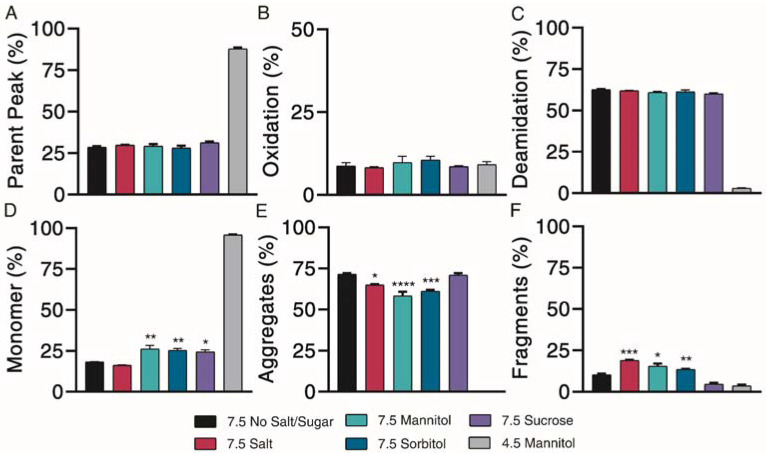
Kinetics of degradation determined by reverse-phase and size exclusion chromatography, with reverse-phase liquid chromatography separating (**A**) parent peak from (**B**) oxidation and (**C**) deamidation chemical impurities and size exclusion chromatography separating (**D**) monomer from (**E**) aggregate and (**F**) fragment physical impurities of exenatide reconstituted at pH 4.5 with mannitol and pH 7.5 with specified excipients after incubation at 37 °C for 4 weeks. (*n* = 3, mean ± SEM, * *p* < 0.05 ** *p* < 0.01 *** *p* < 0.001 **** *p* < 0.0001).

Chemical degradation profiles, in terms of parent peak (Figure 5A) and oxidation (Figure 5B) and deamidation (Figure 5C) chemical impurities were investigated over the course of 4 weeks of incubation at 37 °C. Representative 4-week chromatograms are shown in Supplementary Material Figure S2A. Our results indicated that the addition of different excipients had limited effects on exenatide’s chemical stability at pH 7.5. Relative to the negative control, where 87.8 ± 0.8% of the parent peak remained intact, all pH 7.5 formulations had significant parent peak loss, decreasing to approximately 30% after 4 weeks of incubation, while exhibiting similar levels of oxidation. Similar changes of AUC total, decreasing to an average between approximately 60 and 80% at 4 weeks relative to day 0, indicated some precipitation (Supplementary Material Figure S2B). No noticeable differences were observed between the different formulations.

Physical degradation profiles, in terms of monomers (Figure 5D) and aggregate (Figure 5E) and fragment (Figure 5F) physical impurities were investigated over the course of 4 weeks of incubation at 37 °C. Significant monomer loss was observed for all pH 7.5 formulations, decreasing to approximately 20%. Monomer loss was significantly greater for the pH 7.5 formulation without excipient (18.3 ± 0.1%) than for formulations containing mannitol (26.3 ± 2.0%), sorbitol (25.4 ± 1.1%) and sucrose (24.4 ± 1.3%) (*p* < 0.05 for all solutions), indicating some minor protective abilities of these excipients against physical degradation. Physical degradation of exenatide at pH 7.5 in the presence of various excipients was mainly driven by aggregation.

Further characterization of peptide aggregates was performed using dynamic light scattering. Our data showed that exenatide was stable for the negative control, with particles remaining in the 0.3–10 nm size range over the incubation. At pH 7.5, mannitol, sorbitol and sucrose exhibited stabilizing effects, where 100% of particles were retained in the 0.3–10 nm size range after 4 weeks of incubation (Table 2). On the other hand, the NaCl formulation resulted in a complete shift, where all particles were observed in the 10+ nm range after 4 weeks, indicating that the addition of salt facilitates oligomerization. 

**Figure 6:**
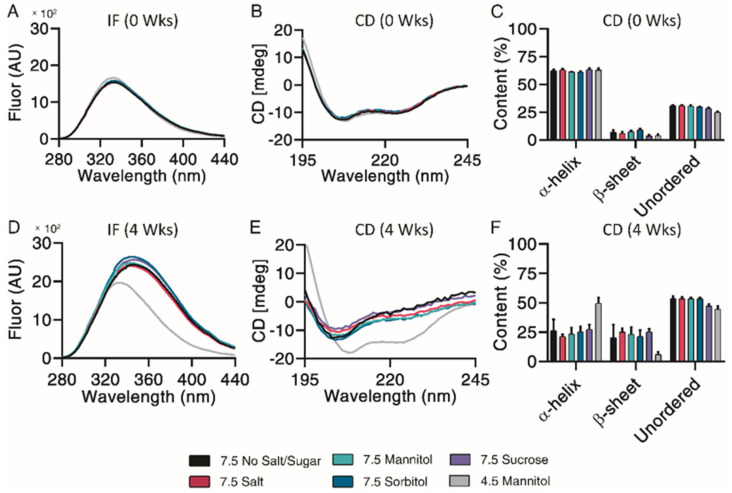
Structural and conformational changes characterized and measured by intrinsic fluorescence (**A**,**D**) and circular dichroism (**B**,**E**), with secondary structure quantified by CDPro analysis (**C**,**F**) for exenatide that was reconstituted at pH 4.5 with mannitol and pH 7.5 with specified excipients after incubation at 37 °C for 4 weeks. (*n* = 3, mean ± SEM).

Subsequent structural analysis was performed by intrinsic fluorescence and circular dichroism. All pH 7.5 formulations experienced a large red shift of maximum emission wavelength and increase in maximum fluorescence intensity after 4 weeks, indicative of Trp-cage unfolding (Figure 6A,D). CD Pro analysis showed no observable differences over the incubation (Figure 6B,E). All pH 7.5 formulations contained similar percentages of α-helix, β-sheet, and unordered content both initially and after 4 weeks of incubation (Figure 6C,F). Taken together, mannitol, sorbitol and sucrose exhibited a minor protective ability to prevent monomer loss and control particle sizes.

## 4. Discussion

The goals of this study were to identify and characterize exenatide’s chemical and physical degradation using accelerated stability testing to elucidate exenatide’s potential underlying degradation mechanisms. To do so, we developed and implemented several orthogonal analytical techniques to investigate the impact of pH and the addition of excipients at elevated pH (NaCl, mannitol, sorbitol, and sucrose) over the course of long-term incubation at 37 °C. Chemical impurities were investigated using reverse-phase liquid chromatography and identified by liquid chromatography-mass spectrometry (QToF). Physical impurities were analyzed by size-exclusion chromatography, with a subsequent examination of particle size distributions by dynamic light scattering. Tertiary and secondary structures were analyzed by intrinsic fluorescence and circular dichroism. Overall, our study provides a valuable body of information regarding exenatide degradation that can be potentially useful for the approval of generic exenatide versions and the development of novel sustained-release formulations.

Over the course of incubation, exenatide appeared to undergo a rapid, pH-dependent degradation at pH 6.5 and above while remaining relatively stable at lower pH. Analogously, endogenous GLP-1 has also shown degradation when pH was slightly increased, though pH was adjusted on a smaller scale [29]. While the loss of exenatide’s parent peak and monomer are pH-dependent, impurity formation varied, with mainly oxidation and fragmentation occurring at pH 6.5 and deamidation and aggregation occurring at pH 7.5 and 8.5. Particle size distributions seemed to further confirm these relationships, indicating size distribution shifts toward the formation of oligomers at higher pH values. Additionally, trends for structural degradation of exenatide reconstituted at an elevated pH seemed to match, indicated by Trp-cage unfolding, a decrease in α-helical content and an increase in unordered content. Exenatide reconstituted at pH 6.5 and 8.5 had greater amounts of β-sheet formation than exenatide reconstituted at pH 7.5, again showing differences of degradation between different pHs. Though not statistically significant, we observed a trend of losses of total AUC after 4 weeks of incubation for exenatide reconstituted at pH 6.5–8.5, indicating precipitation that was further confirmed by shifting of particle sizes. Exenatide chemical impurities were identified by mass shifts from exenatide’s reported average mass (4186) using liquid chromatography-mass spectrometry (QToF), revealing several deamidations (+1 mass shift), oxidations (+16/+32 mass shifts) oxidation/deamidation mixtures (+17 mass shift) and pyro-Q and deamidation impurity (−17 mass shift). However, we have limited knowledge of the actual amino acid residue location of these modifications, as exenatide contains two potential deamidation and two potential oxidation sites. We identified three deamidation impurities (mass shift +1), with one major peak that rapidly increases at high pH. We speculate that the major deamidation impurity occurs on asparagine (N28) as this amino acid is followed by glycine (G29), a prominent deamidation hot spot combination [30,31]. In addition, different deamidation peaks could be attributed to different peptide conformations.21 In terms of oxidation, an impurity that has not been previously studied, we identified a +32 mass shift that could be attributed to either a single oxidation of two residues or a double oxidation of one residue. Additional mass spectrometry analysis of isolated impurity peaks is needed to confirm identities.

Our experiments investigating the impact of different excipients on exenatide’s stability at pH 7.5 revealed the limited ability of excipients to stabilize exenatide as well as their varied effects on exenatide’s degradation profiles. Of the excipients tested, only mannitol, sorbitol and sucrose had minor protective effects against physical degradation, where they slightly prevented monomer loss. Additionally, these excipients appeared to prevent particle size-shifting and even appeared to prevent oligomer formation relative to the formulation without excipient and with salt. Even so, these excipients were unable to prevent chemical degradation. The main impurities observed were aggregation and deamidation for all formulations after 4 weeks of incubation. 

**Figure 7:**
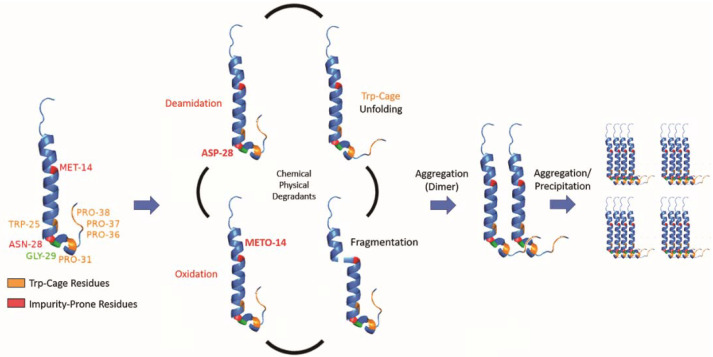
Schematic of proposed exenatide degradation, proceeding through a combination of chemical and physical degradations that includes N28 deamidation (at high pH), M14 oxidation, Trp-cage unfolding, and fragmentation. Degradation is followed by aggregation (dimerization), oligomer formation, and precipitation.

While we determined that exenatide’s degradation kinetics were pH-dependent, we have not fully defined the mechanism of peptide aggregation. Currently, we propose that degradation occurs through a combination of chemical and physical degradation (Figure 7). At high pH, exenatide mainly undergoes N28 deamidation and Trp-cage unfolding that seem to precede further physical degradation, including dimerization, the formation of larger aggregates (oligomers) and precipitation. We also see M14 oxidation in exenatide’s helical region occurring, which could potentially lead to α-helix disruption and unfolding, fragmentation and aggregation. The reactions of deamidation, oxidation and fragmentation are not reversible. However, we observed reversible and concentration-, pH- and temperature-dependent aggregation of exenatide during the preparation of solutions by size exclusion chromatography and dynamic light scattering. For example, we observed that exenatide solutions freshly prepared at room temperature show the presence of dimers by gel permeation chromatography. These dimers appear to dissociate during 30 min incubation at 37 °C as the peptide reaches a conformational equilibrium (data not shown). We also observed the initial presence of oligomers in peptide solutions of pH 4.5 (Table 1), these oligomers appear to dissociate with one-week incubation at 37 °C. This highlights the importance of consistency in sample preparation prior to analysis for data interpretation. In addition, considering the recently published FDA guidance for synthetic peptides, and previously published literature about exenatide’s ability to elicit immunogenic responses, further investigation of the impact of formed exenatide impurities on immunogenicity may be warranted [2,18,32].

## Figures and Tables

**Table 1:** Particle size distribution was determined by dynamic light scattering (by volume) and separated into 0.3–10, 10–100, 100–500, 500–1000 and 1000+ nm size ranges for exenatide that was reconstituted at pH 4.5–8.5 after incubation at 37 °C for 4 weeks; (*n* = 3, mean).

DLS (by Volume)		0.3–10 nm	10–100 nm	100–500 nm	500–1000 nm	1000+ nm
4.5	0 Weeks	77.8	3.5	18.6	0.1	0
	1 Week	100	0	0	0	0
	2 Weeks	100	0	0	0	0
	4 Weeks	100	0	0	0	0
5.5	0 Weeks	100	0	0	0	0
	1 Week	100	0	0	0	0
	2 Weeks	100	0	0	0	0
	4 Weeks	77.8	0	8.8	13.3	0.1
6.5	0 Weeks	100	0	0	0	0
	1 Week	55.6	11.1	28.2	5.1	0
	2 Weeks	88.9	0	1.8	9.3	0
	4 Weeks	33.3	32.5	20.6	13.5	0
7.5	0 Weeks	100	0	0	0	0
	1 Week	88.9	0	9.4	1.8	0
	2 Weeks	100	0	0	0	0
	4 Weeks	33.4	25.2	36.1	5	0.4
8.5	0 Weeks	100	0	0	0	0
	1 Week	44.4	0	54.5	0.9	0.2
	2 Weeks	66.7	29.5	3.8	0	0
	4 Weeks	0	0	27.4	70.1	2.5

**Table 2:** Particle size distribution determined by dynamic light scattering (by volume) and separated into 0.3–10, 10–100, 100–500, 500–1000 and 1000+ nm size ranges for exenatide that was reconstituted at pH 4.5 with mannitol and pH 7.5 with specified excipients when incubated at 37 °C after weeks; (*n* = 3, mean).

DLS (by Volume)	0.3–10 nm	10–100 nm	100–500 nm	500–1000 nm	1000+ nm
4.5 Mannitol	100	0	0	0	0
7.5 No Salt/Sugar	66.6	0	19.8	13.6	0
7.5 NaCl	0	41.9	37.5	20.6	0
7.5 Mannitol	100	0	0	0	0
7.5 Sorbitol	100	0	0	0	0
7.5 Sucrose	100	0	0	0	0

## Supplementary Materials

The following are available online at https://www.mdpi.com/article/10.3390/pharmaceutics13081263/s1, Figure S1. (A) Reverse phase liquid chromatography chromatograms after 4 weeks of incubation, (B) AUCs (relative to day 0), (C) size exclusion chromatography chromatograms after 4 weeks of incubation, (D) AUCs (relative to day 0) for exenatide samples reconstituted at pH 4.5–8.5 over the course of incubation at 37 °C for 4 weeks. Figure S2. (A) Reverse phase liquid chromatography chromatograms after 4 weeks of incubation, (B) AUCs (relative to day 0), (C) size exclusion chromatography chromatograms after 4 weeks of incubation, (D) AUCs (relative to day 0) for exenatide samples reconstituted at pH 4.5 with mannitol vs pH 7.5 with specified excipients over the course of incubation at 37 °C for 4 weeks.
