# Supplementary material for: The Effects of pH and Excipients on Exenatide Stability in Solution
Source: Pharmaceutics. 2021 Aug 16;13(8):1263. doi: 10.3390/pharmaceutics13081263 (PMC8398870; doi:10.3390/pharmaceutics13081263)
Supplement: Supplementary file 1 [file pharmaceutics-13-01263-s001.zip › pharmaceutics-1283820-supplementary.pdf]

pharmaceutics

MDPI

Supplementary Material: The Effects of pH and Excipients on Exenatide Stability in Solution

Alexander Benet, Troy Halseth, Jukyung Kang, April Kim, Rose Ackermann, Santhanakrishnan Srinivasan, Steven Schwendeman and Anna Schwendeman

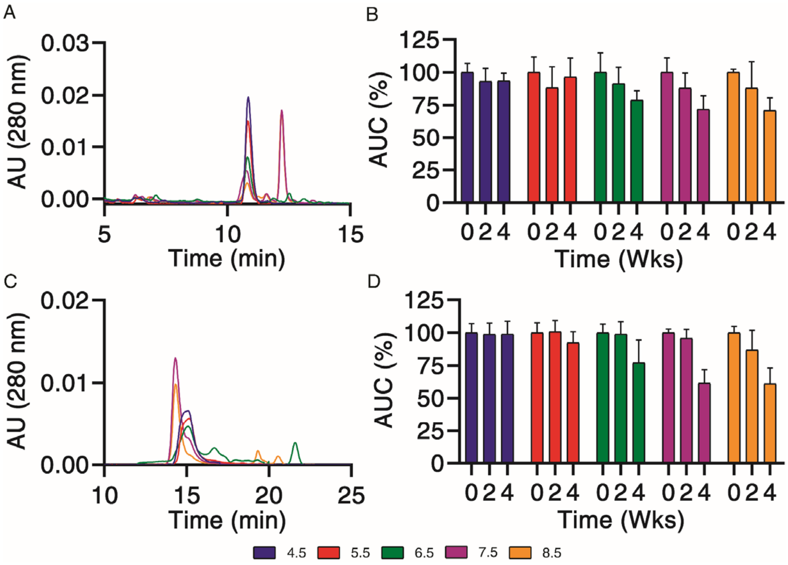

Figure S1. A) Reverse phase liquid chromatography chromatograms after 4 weeks of incubation, **B**) AUCs (relative to day 0), **C**) size exclusion chromatography chromatograms after 4 weeks of incubation, **D**) AUCs (relative to day 0) for exenatide samples reconstituted at pH 4.5-8.5 over the course of incubation at 37 °C for 4 weeks.

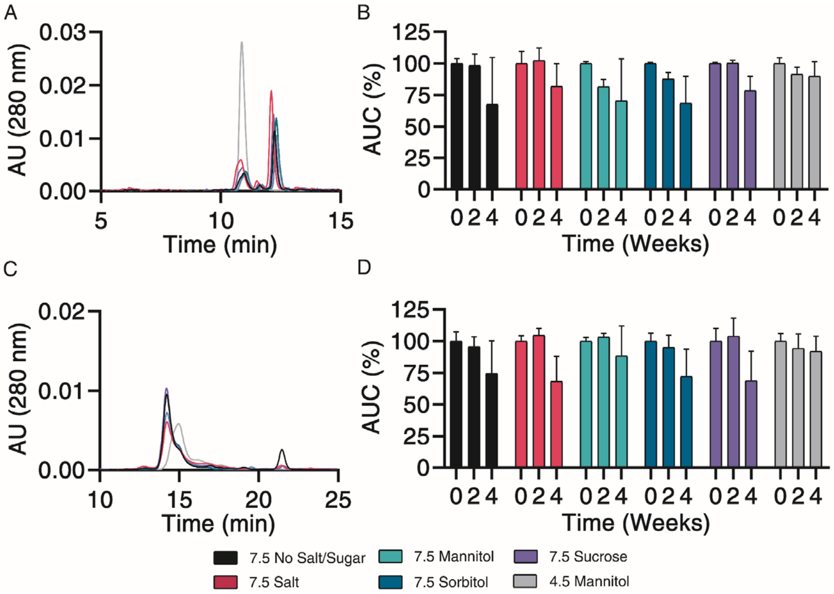

Figure S2. A) Reverse phase liquid chromatography chromatograms after 4 weeks of incubation, **B)** AUCs (relative to day 0), **C**) size exclusion chromatography chromatograms after 4 weeks of incubation, **D**) AUCs (relative to day 0) for exenatide samples reconstituted at pH 4.5 with mannitol vs pH 7.5 with specified excipients over the course of incubation at 37 °C for 4 weeks.
